# Editorial: Advanced imaging and its application in biology and medicine

**DOI:** 10.3389/fcell.2023.1163210

**Published:** 2023-03-08

**Authors:** Veronika Huntosova, Marco Andreana, Angelika Unterhuber

**Affiliations:** ^1^ Center for Interdisciplinary Biosciences, Pavol Jozef Šafárik University in Košice, Košice, Slovakia; ^2^ Center for Medical Physics and Biomedical Engineering, Medical University of Vienna, Vienna, Austria

**Keywords:** live imaging, spectroscopy, molecular biolgy, biophysics, artificial inteligence-AI

The emerging field of advanced imaging technologies in biology and medicine is paving the way for novel applications. Advanced imaging employs sophisticated detection schemes that combine one or more complementary imaging modalities with cutting-edge image analysis. Such multimodality leads to significant improvement in data interpretation and new possibilities of real-time applications in biology and medicine. The aim of this Research Topic is to shed light on recent progress and trends in the development of imaging methods and approaches in biomedicine.

Imaging of blood vessels may reveal significant vascular dysfunction. In particular, the hypoxic state affects not only red blood cells and haemoglobin oxygen saturation but also endothelial cell dysfunction. Monitoring of altered cellular metabolism can be performed by label-free methods such as atomic force microscopy and Raman imaging of specific proteins or by *in vivo* imaging of complex vessels and nerves using computed tomography and widefield imaging. Relaxation and contraction dynamics of vessels and nerves can be monitored by specific labelling methods after intravenous administration of a contrast agent that can be easily imaged. However, detection of specific biomarkers indicating changes in cancer tissue metabolism is often done by immunostaining. With the advanced technique of multiplex immunohistochemistry, multiple parameters can be used to generate large image datasets. These datasets from different modalities can be further analyzed using machine learning methods, leading to new predictive tools for bioimaging.

The current Research Topic in Frontiers in Cell and Developmental Biology includes six articles from 58 authors, covering a wide range of advanced imaging technologies, analysis approaches, and applications. The authors have shed light on the use of advanced imaging approaches for recent and ongoing research efforts in biology and medicine. A total of five original research and one data report articles in this research area have been accepted. The collection encompasses different areas of investigations as further detailed by the sections below.

Cellular hypoxia has been hypothesized to play an important role in the ageing process. Endothelial cell dysfunction may be responsible for the development of various diseases. Hypoxic conditions acting on cells can change their phenotype to senescence and affect the expression of vascular endothelial growth factor in these cells. Angom et al. investigated nanomechanical changes in the membrane and analyzed in detail the morphological and structural changes of endothelial cells by atomic force microscopy under hypoxia-induced senescence. Their work identified vascular endothelial growth factor and Yes-associated protein of the Hippo pathway as important in the signaling pathways in this condition. These signaling pathways and nanomechanical changes in endothelial cells may therefore be important targets in age-related disease models.

Cytochrome c redox balance plays an important role in mitochondrial state and cellular respiration. Abramczyk et al. studied the oxidized and reduced forms of cytochrome c in sperm cells using Raman imaging. They identified cytochrome c as the key molecule that determines the life and death of human male reproductive cells. In this work, Raman imaging represents a label-free technique that has been shown to be a useful tool for rapid fertility testing based on the redox status of mitochondria.

Neuro-modulation strategies for peripheral nerves could reduce the extensive use of pharmacological drugs that cause adverse effects in patients. Vagus nerve stimulation may be useful in cardiovascular medicine. Proper knowledge about the morphology of the cardiac vagus nerve is important for selective stimulation. Kronsteiner et al. used microcomputed tomography and three-dimensional reconstructions to visualize the topography and anatomy of cardiac vagal autonomic innervation in rabbits and pigs from the cervical level down to the heart. Their findings on species-specific cardiac branching highlighted the need for further characterization of cardiac autonomic innervation and new *in silico* models to improve stimulation patterns for selective cardiac vagus nerve stimulation.

Blood vessels and nerves should be coordinated in the proper erectile function of male reproductive organs. Therefore, the penile microcirculatory system and erectile responses might be related. Fujimoto et al. evaluated relaxation/contraction dynamics by imaging a new parameterized model of erectile dysfunction. They visualized analyses of sinusoidal dynamics in the mouse model using Patent Blue dye. The model system they studied provides unique information to further investigate the regulation of erectile dysfunction.

Deep learning algorithms are machine learning techniques that have attracted much interest in recent years due to the rapid detection of selective patterns. Zhao et al. developed an algorithm to predict haemoglobin concentration and screen for anemia using ultra-widefield fundus images in large datasets of patient samples. The proposed algorithm focuses on the blood vessels in the optic nerve head and macular region with special attention given to the vessels in the periphery of the fundus. The distribution of peripheral vessels is an important parameter than can be enhanced with the deep learning mode. It provides a comprehensive, sensitive, and non-invasive approach for screening and predicting anemia in a large population study.

Cancer exhibits different pathological features and metabolic landscapes at different stages, so liver cancer can be diagnosed using serological tests, diagnostic imaging, and histology. Multiplex immunohistochemistry provides a tool to analyze multiple biomarkers in a single tissue section. The work of Jiang et al. represents the comprehensive study of cell composition, cell functions, and cell-cell interactions *in situ*. Samples from a liver cancer were analyzed using multiplex staining and then evaluated using machine learning methods for tissue segmentation, cell segmentation, and phenotype.

In summary, this Research Topic gathered a wide spectrum of high-quality articles demonstrating the impact of advanced imaging in the fields of biology and medicine. We acknowledge all authors who contributed to this Research Topic, and we hope that this Research Topic will serve to inspire the scientific community by providing new routes for understanding biological process and human diseases.

